# The ability of locked nucleic acid oligonucleotides to pre-structure the double helix: A molecular simulation and binding study

**DOI:** 10.1371/journal.pone.0211651

**Published:** 2019-02-12

**Authors:** You Xu, Olof Gissberg, Y. Vladimir Pabon-Martinez, Jesper Wengel, Karin E. Lundin, C. I. Edvard Smith, Rula Zain, Lennart Nilsson, Alessandra Villa

**Affiliations:** 1 Department of Biosciences and Nutrition, Karolinska Institutet, Huddinge, Sweden; 2 Department of Laboratory Medicine, Karolinska Institutet, Huddinge, Sweden; 3 Biomolecular Nanoscale Engineering Center, Department of Physics, Chemistry and Pharmacy, University of Southern Denmark, Odense M, Denmark; 4 Department of Clinical Genetics, Centre for Rare Diseases, Karolinska University Hospital, Stockholm, Sweden; Wake Forest University, UNITED STATES

## Abstract

Locked nucleic acid (LNA) oligonucleotides bind DNA target sequences forming Watson-Crick and Hoogsteen base pairs, and are therefore of interest for medical applications. To be biologically active, such an oligonucleotide has to efficiently bind the target sequence. Here we used molecular dynamics simulations and electrophoresis mobility shift assays to elucidate the relation between helical structure and affinity for LNA-containing oligonucleotides. In particular, we have studied how LNA substitutions in the polypyrimidine strand of a duplex (thus forming a *hetero* duplex, *i*.*e*. a duplex with a DNA polypurine strand and an LNA/DNA polypyrimidine strand) enhance triplex formation. Based on seven polypyrimidine single strand oligonucleotides, having LNAs in different positions and quantities, we show that alternating LNA with one or more non-modified DNA nucleotides pre-organizes the *hetero* duplex toward a triple-helical-like conformation. This in turn promotes triplex formation, while consecutive LNAs distort the duplex structure disfavoring triplex formation. The results support the hypothesis that a pre-organization in the *hetero* duplex structure enhances the binding of triplex forming oligonucleotides. Our findings may serve as a criterion in the design of new tools for efficient oligonucleotide hybridization.

## Introduction

Nucleic acid hybridization plays a key role in biotechnological and medical applications [1,2]. An effective approach is to design oligonucleotides (ONs) that bind a DNA target sequence with high affinity and specificity, using both Watson-Crick (WC) and Hoogsteen (HG) base pairing. To competitively bind an ON to a target, the binding affinity needs to be higher than in the original (DNA) duplex. This can be achieved by using synthetically modified nucleotides, for instance having a modified sugar moiety that restricts backbone and ribose conformational flexibility as in locked nucleic acid (LNA) [3–5].

**Fig 1:**
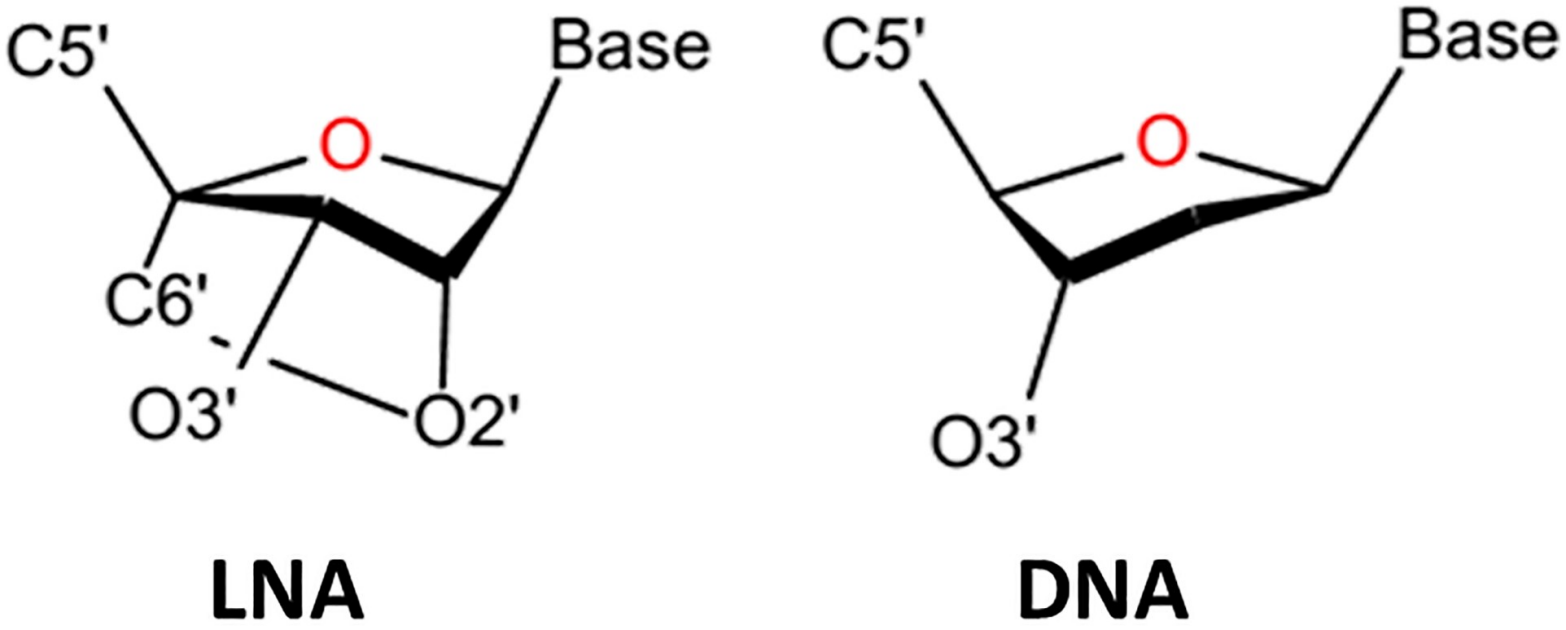
*North* and *south* conformation of the ribose and deoxyribose in LNA and DNA, respectively.

LNAs are characterized by having a methylene group bridging atoms 2’ oxygen and C4’ of the sugar ring (Fig 1) locking the ribose in the *C3*’*endo* or *north* configuration (a non-modified deoxynucleotide exists typically either in the *C2*’*endo*/*south* or the *C3*’*endo/north* conformations). The presence of LNA in an ON enhances its binding affinity to a DNA complementary strand and induces an A-form helical conformation [6,7]. Molecular dynamics (MD) studies have shown that the double helix slightly unwinds when LNA is introduced in either strand [8,9] and such an under-wound conformation is characterized by having negatively shifted slide and twist [9,10]. Alternating DNA and LNA residues in triplex forming oligonucleotides (TFOs) also significantly increases the melting temperature [3,11,12]. Thus, LNA-containing ONs show strong binding affinity for endogenous nucleic acids, which provides the opportunity to efficiently target such sequences. Those facts highlight LNA as a potential candidate in gene regulation and promote future studies with biotechnological and *in vivo* applications.

In a recent study, we have shown that LNA substitutions in the polypyrimidine strand of a duplex (thus forming a *hetero* duplex, *i*.*e*. a duplex with a DNA polypurine strand and an LNA/DNA polypyrimidine strand) enhance triplex formation[12]. In particular, alternating LNA and DNA nucleotides shifts the double helical conformation from a B-like towards an A-like helix and favors triplex formation. However, the experimental data reported from *hetero* duplexes and corresponding triplexes were limited and therefore more detailed predictions are needed.

Here, we investigate in a systematic way the ability of LNA to pre-structure the double helix and to promote TFO binding. To achieve this, we used MD simulation to address LNA-induced structural features and electrophoresis mobility shift assays (EMSA) to quantify TFO binding to different duplex targets under various conditions. MD simulations have successfully been used to investigate LNA-containing double and triple helix structures[8,9,12,13]. The CHARMM force field has recently been calibrated to enable investigation of the structural influence of LNA in RNA and DNA duplexes[14]. Seven polypyrimidine strands with different amount and position of LNA nucleotides, but targeting the same polypurine strand, have been studied (Table 1), and an alternating DNA/LNA single-strand oligonucleotide yielding potent hybridization was used as TFO. The simulated helical structures have been compared with a reference triplex structure, allowing the selection of four ONs (Table 2) for binding experiments. In all cases, TFO binding was assessed in the presence or absence of the triplex-specific intercalating compound, benzoquinoquinoxaline (BQQ). [15,16]

**Table 1:** Polypurine (R), polypyrimidine (Y) and TFO (T) sequences used in simulated oligonucleotides. The *hetero* duplexes and triplexes are named after the polypyrimidine strand. Underlined residues are LNAs and shaded region indicates TFO-binding domain.

Oligonucleo-tide (ON)		Sequence			Duplex (R:Y)	Triplex (R:Y·T)
R	DNA	5’	GGGGAAAAGAAAAAAGA	3’		
T	1D1L	5’	CCTTTTCTTTTTTCT	3’		
Y	DNA	5’	TCTTTTTTCTTTTCCCC	3’	dupDNA (DNA:DNA)	trpDNA (DNA:DNA·1D1L)
	1D1L		$\underline{\text{T}}\text{C}\underline{\text{T}}\text{T}\underline{\text{T}}\text{T}\underline{\text{T}}\text{T}\underline{\text{C}}\text{T}\underline{\text{T}}\text{T}\underline{\text{T}}\text{C}\underline{\text{C}}$CC		dup1D1L (DNA:1D1L)	trp1D1L (DNA:1D1L·1D1L)
	2D1L		$\underline{\text{T}}\text{CT}\underline{\text{T}}\text{TT}\underline{\text{T}}\text{TC}\underline{\text{T}}\text{TT}\underline{\text{T}}\text{CC}$CC		dup2D1L (DNA:2D1L)	trp2D1L (DNA:2D1L·1D1L)
	3D1L		$\text{T}\underline{\text{C}}\text{TTT}\underline{\text{T}}\text{TTC}\underline{\text{T}}\text{TTT}\underline{\text{C}}\text{C}$CC		dup3D1L (DNA:3D1L)	trp3D1L (DNA:3D1L·1D1L)
	2D2L		$\text{T}\underline{\text{CT}}\text{TT}\underline{\text{TT}}\text{TC}\underline{\text{TT}}\text{TT}\underline{\text{CC}}$CC		dup2D2L (DNA:2D2L)	trp2D2L (DNA:2D2L·1D1L)
	2L2D		$\underline{\text{TC}}\text{TT}\underline{\text{TT}}\text{TT}\underline{\text{CT}}\text{TT}\underline{\text{TC}}\text{C}$CC		dup2L2D (DNA:2L2D)	trp2L2D (DNA:2L2D·1D1L)
	D5LD		$\text{TCTTT}\underline{\text{TTTCT}}\text{TTTCC}$CC		dupD5LD (DNA:D5LD)	trpD5LD (DNA:D5LD·1D1L)
	LNA		$\underline{\text{TCTTTTTTCTTTTCC}}$CC		dupLNA (DNA:LNA)	trpLNA (DNA:LNA·1D1L)

**Table 2:** Polypurine (R), polypyrimidine (Y) and TFO (T) sequences used in the EMSA experiments. The *hetero* duplexes are named after the polypyrimidine strand. Underlined residues are LNAs and shaded region indicates TFO-binding domain.

Oligonucleotide (ON)		Sequence				
T	1D1L	5’		CCTTTTCTTTTTTCT		3’
R	DNA	5’	AGCAGAGGGCGTGGG	GGAAAAGAAAAAAGA	TCCACCGGTCGCCAC	3’
Y	45-DNA	5’	GTGGCGACCGGTGGA	TCTTTTTTCTTTTCC	CCCACGCCCTCTGCT	3’
	45-1D1L		GTGGCGACCGGTGGA	TCTTTTTTCTTTTCC	CCCACGCCCTCTGCT	
	45-2L2D		GTGGCGACCGGTGGA	TCTTTTTTCTTTTCC	CCCACGCCCTCTGCT	
	45-D5LD		GTGGCGACCGGTGGA	TCTTTTTTCTTTTCC	CCCACGCCCTCTGCT	
	45-2D1L		GTGGCGACCGGTGGA	TCTTTTTTCTTTTCC	CCCACGCCCTCTGCT	

## Methods

### Molecular dynamics (MD) simulations

#### System setup

The 17-mer *hetero* duplex and the corresponding triplex structures were generated using the 3DNA web server[17]. The duplexes were built in a B-DNA conformation and all triplexes were based on a T:A·T DNA (“:” and “·” denote Watson-Crick and Hoogsteen base pairing, respectively) fiber model.[18] The bases of the triplex were then modified to correspond to the target sequence. DNA nucleotides in the polypyrimidine strand and in the TFO were substituted by LNA by patching a methylene group on the ribose, and all cytidines with LNA were 5-methylated (m^5^C). The LNA sugars were energy-minimized in 100 steps using the steepest descent method, reaching the *north* pucker.

#### Simulation details

The CHARMM36 force field for nucleic acids[19,20] and modified nucleotides[14,21] was used. The structures were energy-minimized in 500 steps using the Adopted-Basis Newton-Raphson method *in vacuum*, with a harmonic restraint using a force constant of 10 kcal/mol·Å^2^ on backbone atoms and on the distances of base pair hydrogen bonds. All structures were then solvated in a cubic box using theTIP3P water model [22]. The shortest distance between box edge and solute is 8 Å and periodic boundary conditions were applied. The systems were first neutralized by adding sodium ions (Na^+^) and then more Na^+^ and chloride (Cl^-^) ions were added corresponding to a salt concentration of 0.15 M NaCl. The particle mesh Ewald method [23] was applied for long range electrostatic interactions, with a direct space cutoff of 9 Å, and a switch function over the range 8–9 Å was used on the force of van der Waals interactions. The simulations were performed using Langevin dynamics with a friction coefficient of 5 ps^-1^ in the NPT ensemble.

Simulations were performed on graphical processing units with the program CHARMM [24] and the OpenMM interface [25]. The leap-frog integrator with a 2 fs time step was used. Bonds involving hydrogen atoms were constrained using the SHAKE algorithm [26]. Before the production run all systems were equilibrated in 10 ns at 298 K with harmonic restraints on hydrogen bond distances of end base pairs and backbone atoms. The productions were run 500 ns for *hetero* duplexes and triplexes. A harmonic force constant of 10 kcal/mol·Å^2^ with 2.9 Å equilibrium distance between heavy atoms was applied on the hydrogen bonds of end Watson-Crick (WC) base pairs in all duplexes and triplexes.

Snapshots, saved every 50 ps, were analyzed using CHARMM and Curves+ [27] software packages. The first 20 ns of each run have been excluded from the analysis. To check the maintenance of base pairs in the duplex and triplex, the N1-N3 distances of WC base pairs and N7-N3 of Hoogsteen (HG) base pairs were monitored. A distance shorter than 3.5 Å indicates that a hydrogen bond is formed between the heavy atoms and the bases are then considered to be paired. If not specified, the base pair geometries were calculated by excluding the last two nucleotides in each end.

### Oligonucleotides

Mixmer DNA/LNA ONs for the gel shift experiments were 45 bases long forming *hetero* duplexes when hybridized to the 45-polypurine DNA ON. All duplexes contain the TFO target region in the center according to Table 2. LNA containing ONs were synthesized using solid phase phosphoramidite chemistry on an automated DNA synthesizer in 1.0 mmol synthesis scale as previously described.[4] RP-HPLC or IE-HPLC purification (to 85%) was performed, after which the composition of all synthesized ONs was verified using MALDI-MS analysis recorded using 3-hydroxypicolinic acid as a matrix. The 45-mer polypurine and polypyrimidine all-DNA ONs were purchased from Sigma Aldrich. ON concentrations of stock solutions were verified using a Nanodrop spectrophotometer (Thermo Scientific).

#### Oligonucleotide hybridization

The duplex target was pre-annealed by mixing the 45-mer polypurine and polypyrimidine ONs (Table 2) at 1 μM concentration using a slight excess of the polypyrimidine ON in intranuclear buffer (50 mM Tris-acetate, pH 7.4, 120 mM KCl, 5 mM NaCl, 0.5 mM MgOAc). The ONs were heated prior to hybridization to 95°C and slowly cooled to room temperature, after which the TFO was added in concentrations corresponding to different ratios to the duplex target (2:1, 24:1) in the absence or presence of the triplex-intercalating compound BQQ [15], (molar ratio 100:1 relative to the duplex target). The complexes were then incubated at room temperature for 0.5, 1 or 24h in 10 μL total volume intranuclear buffer, and later analyzed using gel electrophoresis. The samples were frozen and stored in -20°C until analyzed.

#### Electrophoretic mobility shift assay (EMSA)

5 μL of the hybridization mixture was loaded on a 20% non-denaturing polyacrylamide gel gel together with 1 μL 6x Orange Loading Dye (Thermo Fisher), and samples were separated at 90 V during 5h in 1x Tris Borate EDTA (TBE) running buffer (Fisher Scientific). After electrophoresis, the gels were stained with SYBR Gold (Thermo Fisher) diluted 1:1000 for 7 minutes, analyzed in a Versadoc instrument (BioRad) equipped with a CCD camera, and the intensity of the gel bands quantified using Quantity One software (BioRad). The amount of triplex formed was calculated from the band intensities and the ratio between the triplex to the total band intensities in each lane. All experiments were conducted three or four times.

## Results and discussion

### *LNA/DNA hetero* duplexes

Substituting DNA nucleotides with LNA improves duplex formation and stability.[28] Recently, we have shown that alternating DNA/LNA nucleotides in the polypyrimidine strand enhances the triplex formation due to pre-structuring of the duplex.[12] Here we investigate where and how many LNAs in the duplex are needed for the best triplex formation: a series of *hetero* duplexes have been designed with the same purine/pyrimidine sequence and length (17 mer) but with diverse LNA substitution patterns in the polypyrimidine strand (Table 1). For the simulations, the corresponding triplexes were built using residues 3 to 17 of the polypurine strand as targets for the TFO binding, and a polypyrimidine strand with alternating LNA and DNA nucleotides as TFO. The previously studied *hetero* duplexes include the DNA duplex with alternating LNA substitution in the polypyrimidine strand (dup1D1L)[12] and the newly designed duplexes include a polypyrimidine strand that contains one LNA for every two or three DNA nucleotides (dup2D1L and dup3D1L, respectively), two consecutive LNAs flanked by two DNA nucleotides (dup2D2L and dup2L2D), five consecutive LNAs flanked by DNAs (dupD5LD) or full LNA ONs (dupLNA). For comparison the corresponding *homo* duplex (dupDNA) was also simulated.

#### LNA position and *hetero* duplex conformation

**Fig 2:**
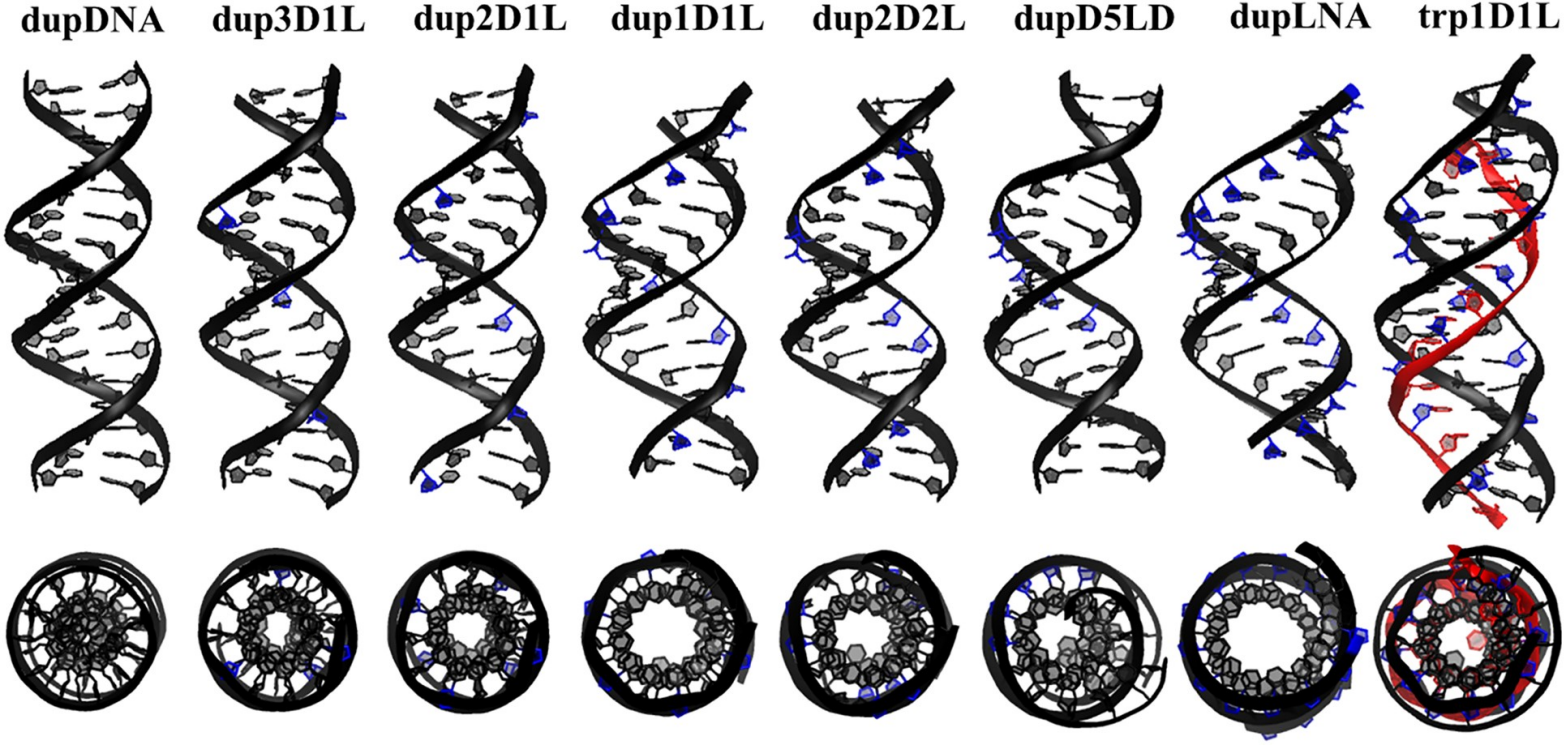
Average structures of the seven simulated duplexes. For comparison the average structure of the triplex trp1D1L is also shown. Duplex strands are in black with LNA sugars in blue, and the TFO is in red. All the structures are aligned on the same plane. ON sequences for both the duplex and triplex structures are presented in Table 1.

**Fig 3:**
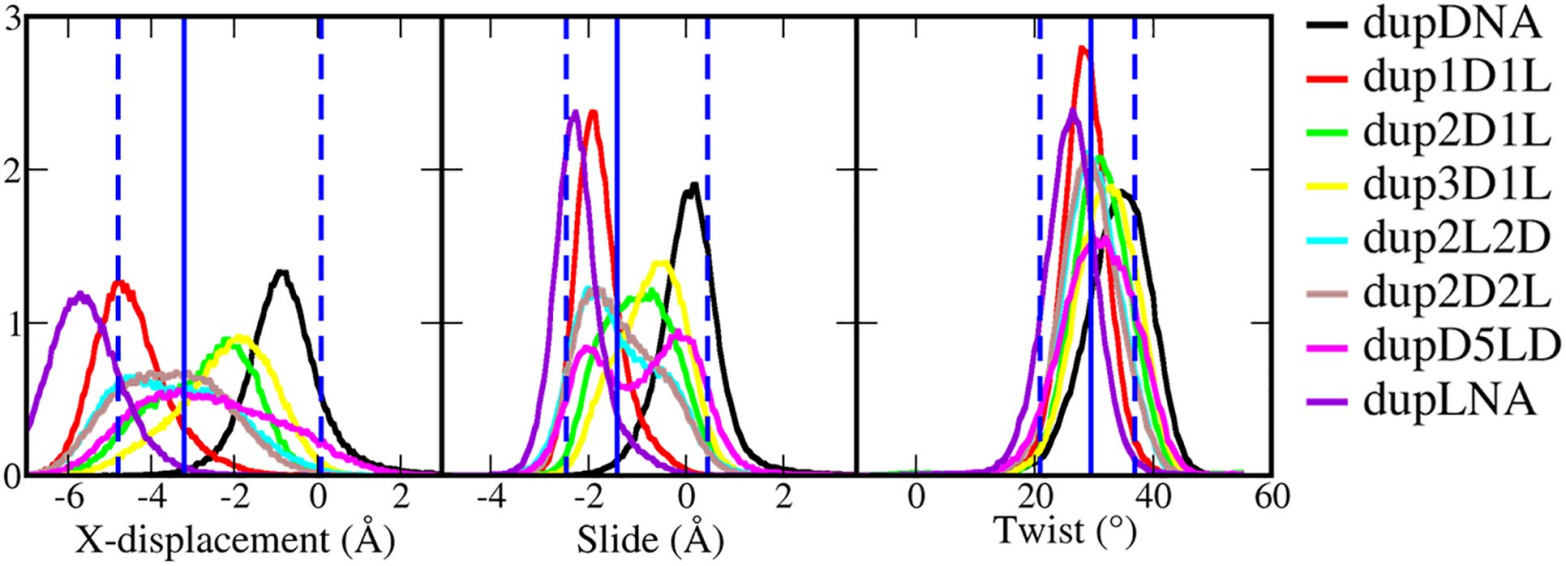
Base pair geometries (x-displacement, slide and twist) for duplex structures: X-displacement, slide and twist. The histograms were sampled from 500 ns simulations. The blue vertical lines show the statistical data from trpDNA and trp1D1L (triple-helix-type reference), where the solid line is the average value and dashed lines indicate the range covering the conformation of 95% snapshots. ON sequences for both the duplex and triplex structures are presented in Table 1.

The *hetero* duplexes were simulated for 500 ns in NaCl solution (0.15 M). All the helical structures were stable (Fig 2) and kept the WC hydrogen bond pattern (S1 Fig). The *homo* duplex, dupDNA, is in normal B-DNA form, while all other duplexes show gradual conformational change toward A-type helix as soon as the number of LNA substitutions increases (Fig 2). The conformations of dup1D1L and dup2D2L are very similar to the duplex structure in the triplex trp1D1L (DNA:1D1L·1D1L), while dup3D1L and dup2D1L show a conformation that is an intermediate between the *homo* duplex dupDNA and the *hetero* duplex dup1D1L, probably due to the lower content of LNA in the polypyrimidine strand than in dup1D1L and dup2D2L. Complete substitution by LNAs in the polypyrimidine strand (dupLNA) unwound the helical structure, which becomes shorter and wider than typical A-DNA, as previously reported[9]. Having consecutive LNAs in the middle of the polypyrimidine strand, as in dupD5LD, results in helical bending, likely owing to the different conformations of the DNA and LNA sections (e.g the bimodal distribution of slide in Fig 3). Neither dupD5LD nor dupLNA are conformationally similar to triplex trp1D1L, and hence they require further structural adaption to form a triple helix structure.

A DNA duplex shifts its B-type conformation toward A-type upon TFO binding: the base pairs in the double helix remain perpendicular to the helical axis but the slide and x-displacement become negative and twist is reduced.[29] In a recent study,[12] we have shown that LNA substitutions in the DNA polypyrimidine strand modulate the duplex helical conformation toward a triple-helix-type, thereby promoting TFO binding. We have named this double helix conformation LirA (low inclination & roll A-DNA). The base pair step helicoidal parameters for the *hetero* duplexes, calculated from a 500 ns simulation, show that the base pair step parameters mostly affected by LNA substitutions, are x-displacement, slide and twist (S1 Table). These three parameters are then used to rate the triplex forming ability of the different duplexes. The hypothesis is that: the more the duplex conformation is similar to a triple-helix-type conformation, the higher is the efficiency to bind a TFO. This means that TFO binding is more favorable when less helical structural adaption of the target duplex is required. To define a reference for a triple-helix-type conformation, we took the average values of x-displacement (-3.2 Å), slide (-1.4 Å) and twist (29.5°) from the trpDNA (DNA:DNA·1D1L) and trp1D1L (DNA:1D1L·1D1L) triplex structures. In general LNA and DNA nucleotides in the polypyrimidine strand influence the x-displacement, slide and twist parameter values in opposite directions: DNA residues have larger base-pair step parameter than the reference, while LNA nucleotides have lower values, as clearly shown by the distributions for the *homo* duplex dupDNA and the *hetero* duplex dupLNA (Fig 3).

Alternating LNA and DNA residues in the polypyrimidine strand generates the duplex with the closest conformation to the triple-helix-type reference (Fig 3). On average, the *hetero* duplexes dup1D1L and dup2D1L have values closer to the reference than the *hetero* duplex dupLNA and the *homo* duplex (dupDNA). Dup1D1L values are decreased and dup2D1L increased compared to the reference; the average x-displacement, slide and twist are -4.5/-2.8 Å, -1.8/-1.0 Å and 29/31° respectively (S1 Table). Dup3D1L has fewer LNAs, so its conformation distribution was further right-shifted than dup2D1L. Changing the 1D1L pattern to 2D2L or 2L2D keeps the same fraction of DNA and LNA nucleotides in the polypyrimidine strand, but generates slightly different conformations: dup2D2L and dup2L2D parameters are characterized by having a wider distribution and an average value (-3.5 Å, -1.3 Å and 29°) closer to the reference than the other duplexes. Increasing the number of consecutive LNAs from two to five (like in D5LD) yielded wider and bimodal distributions for the observed base pair parameters (Fig 3), indicating that ONs containing consecutive LNA nucleotides induce inhomogeneity in the helical geometry.

In summary, except for dupDNA and dupLNA, the conformations of the other duplexes are mostly located in the range of the reference. In terms of average values, dup2D2L and dup2L2L are closest to the reference, and thereafter dup1D1L and dup2D1L, followed by dup3D1L, and finally dupD5LD. However, consecutive flanking LNAs induce inhomogeneity in the helical geometry, and if the triplex conformation is homogeneous, the ability of TFO binding of dup2D2L/2L2D and dupD5LD is probably reduced because of an additional conformational adaption being required.

### *Hetero* triplexes

To get further information regarding the corresponding triplex conformations, we have simulated the duplexes with a TFO in the major groove. A DNA/LNA TFO (Table 1) was used, since it has been shown that flanking DNA with LNA promotes TFO binding [3,12]. Here we wanted to compare helicoidal conformation of the duplexes, when they are in a triple-helix context.

The majority of the triple-helix structures kept most of the WC and HG hydrogen bond patterns (S2 Fig). Fluctuations are observed at the ends of TFOs due to thermal motion of end nucleotides. For trpD5LD and trpLNA, breaking of WC and HG hydrogen bonds are observed (S2 Fig), indicating a low stability of the triple-helix structure. More than two consecutive LNAs in the polypyrimidine strand of the duplex increases the rigidity and inhibits the conformation rearrangement upon TFO binding.

To verify the identified design requirements for an efficient TFO-binding duplex, we have tested the TFO binding affinity for a group of *hetero* duplexes. We have selected *hetero* duplexes that show both minor and major conformational adaption upon TFO binding (a duplex that has similar conformation in the unbound and bound state). No or minor conformational adaption is observed for dup1D1L, dup2D1L, dup2D2L, while dupD5LD is inhomogeneous which might hamper the conformational adaption upon binding.

### Analysis of TFO binding using electrophoretic mobility shift assay (EMSA)

**Fig 4:**
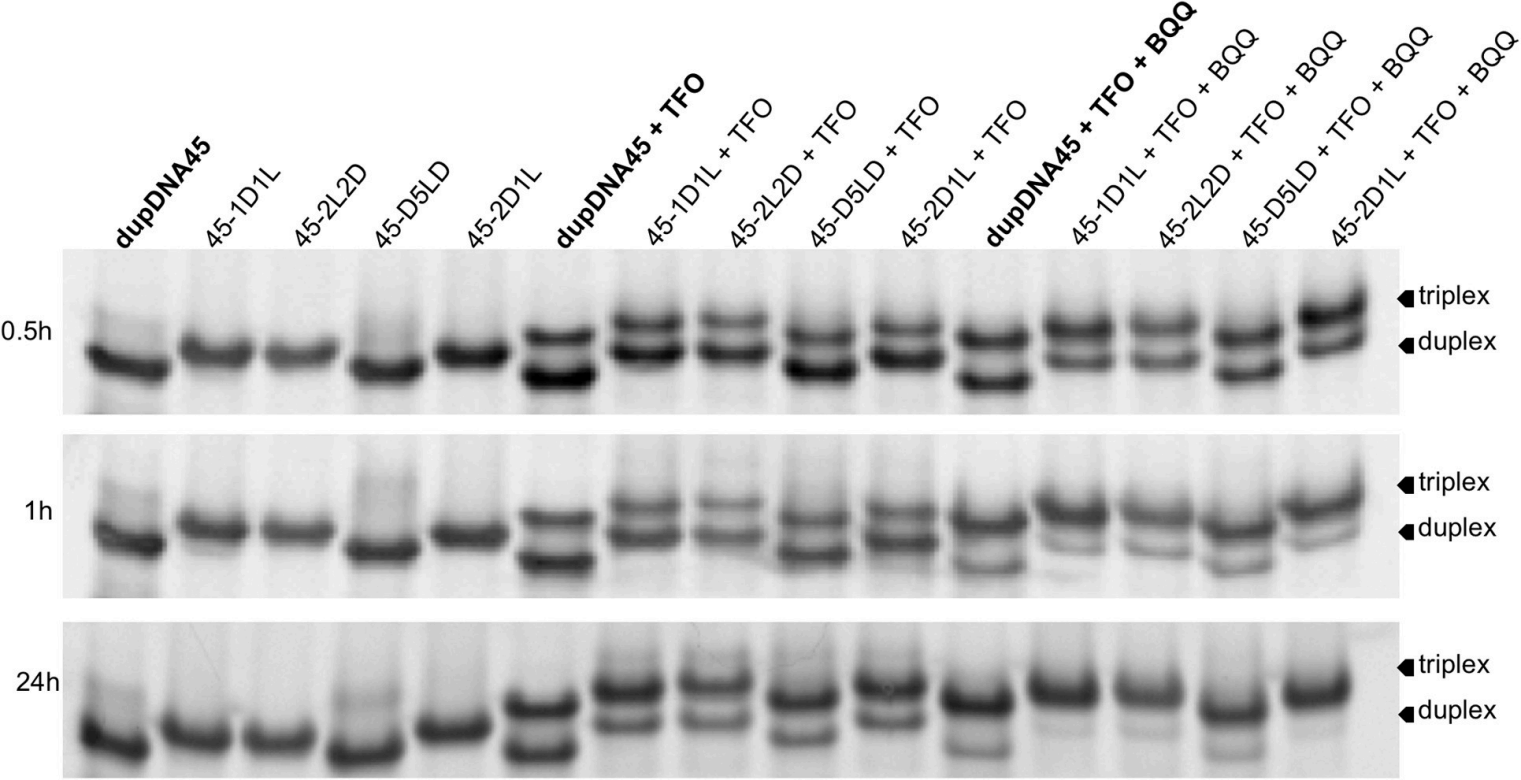
Representative EMSA gel images of TFO binding to the different duplexes. The TFO to duplex target ratio shown is 2:1 in the absence or presence of BQQ (100:1 molar ratio compared to the duplex) at 0.5, 1, and 24 h. The lower mobility band (top band) corresponds to the triplex whereas the higher mobility band (lower band) corresponds to the duplex structure. The *homo* duplex is named dupDNA45 while *hetero* duplexes are named after the polypyrimidine strand.

In order to experimentally assess TFO binding to a duplex target containing various LNA substitutions, a selection from the modeled *hetero* duplexes was tested. The duplexes were incubated with the TFO and analyzed using EMSA (see Table 2 for sequences). The target duplexes were stabilized by adding flanking regions of 15 nucleotides on each side of the TFO binding site to reproduce a more biologically relevant situation (45 nucleotides in total). The time points tested were 0.5, 1 and 24 h using a TFO to duplex ratio of 2:1 or 24:1. In addition, in some samples the triplex-intercalating compound BQQ was added and incubated together with the TFO and the preformed target duplex. In Fig 4, some representative gels can be seen for the TFO to duplex ratio of 2:1 at the three time points. Importantly, at the low TFO to duplex ratios used here it was possible to increase the duplex ON concentrations so we could investigate the TFO to duplex binding without the need for radioactive labeling of the target, which is normally used for this type of assays[12]. This demonstrates the feasibility of using a non-radioactive EMSA, saving time and reducing risks.

**Fig 5:**
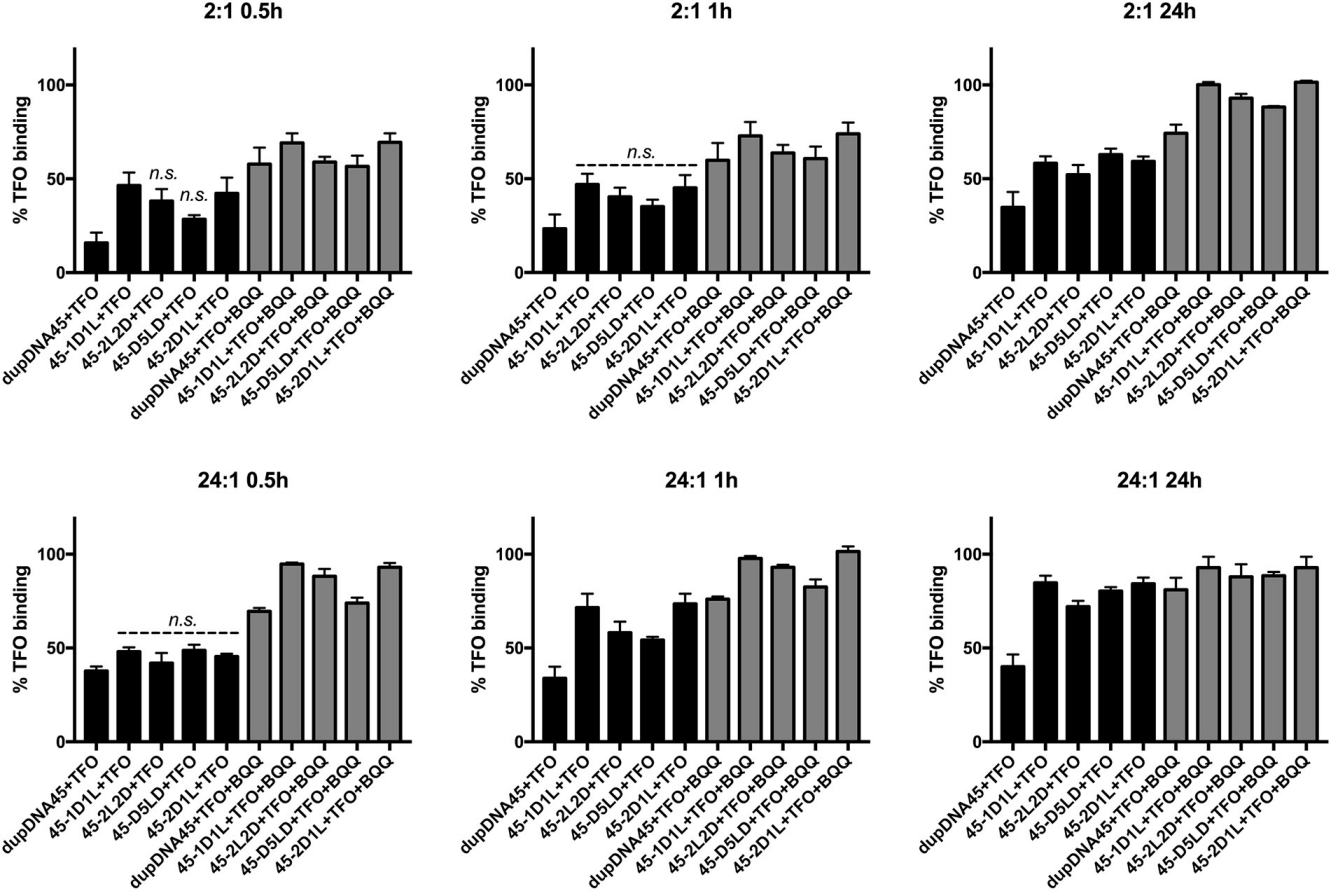
Densitometry quantitation of the EMSA bands. Graphs shows the percentage of TFO binding at 0.5, 1 and 24 h respectively at the two ratios studied (2:1 and 24:1 TFO:duplex). For duplex sequences refer to Fig 4. Non-significant differences compared to the *homo* duplex dupDNA45 + TFO are marked with n.s. All other values are significantly higher compared to dupDNA45 +TFO (p = 0.05). Statistical analysis was performed using GraphPad prism (v6) and one-way ANOVA to determine significance. Error bars show the mean+SEM, n = 4 for all samples and time points, except for the 45-D5LD samples where n = 3.

In Fig 5, the percentage of TFO binding to the target duplexes was quantified for all experiments (n = 3 or 4) at the examined time points and TFO:Duplex ratios. Even though no major differences in percentage of triplex formation could be detected among the *hetero* duplexes, regardless of whether BQQ was present or not, the data suggest a relationship between the number of adjacent LNA residues in the duplex binding region and the TFO binding rate. At the ratio 2:1 (TFO:duplex) the shorter time points do not show significant differences when comparing the *hetero* duplexes with the *homo* duplex, with the exception of 45-1D1L and 45-2D1L *hetero* duplexes. This might suggest a more efficient accommodation of the triplex in 45-1D1L and 45-2D1L compared to the 45-2L2D and 45-D5LD *hetero* duplexes. Even though the small, but favorable trend remains for the 45-1D1L and 45-2D1L, at later time points and higher ratio the results for the *hetero* duplex targets are not significantly different with or without the BQQ being present.

**Fig 6:**
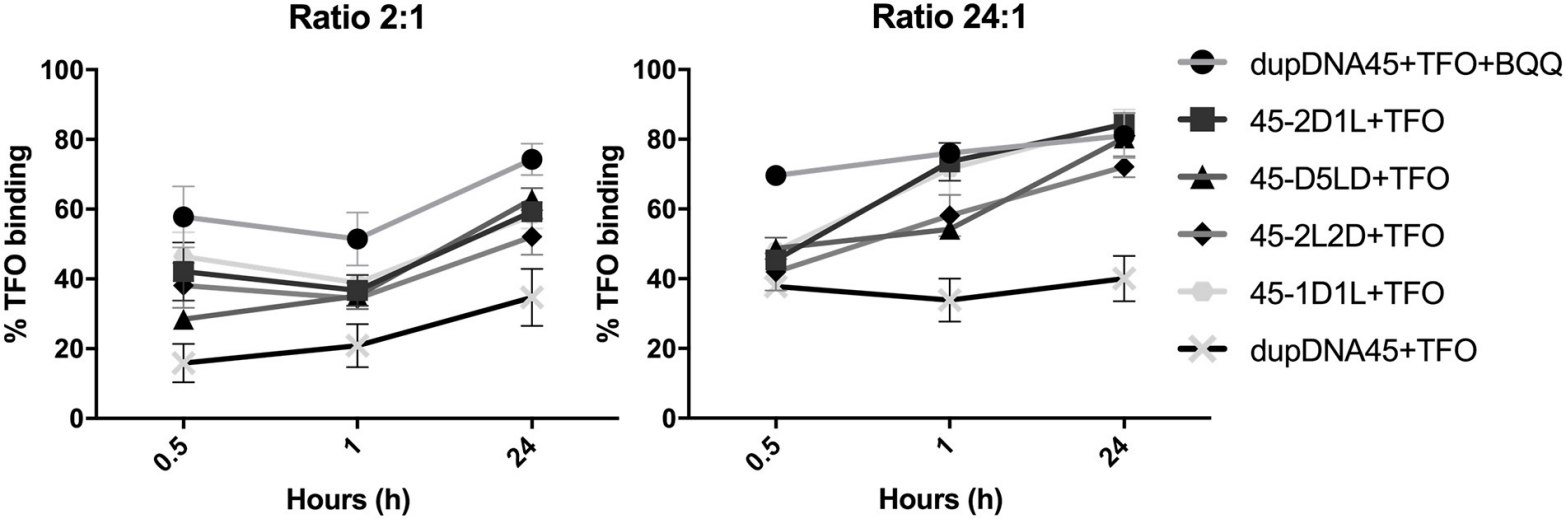
TFO-binding of duplex targets and triplex formation over time. Graphs show *homo* and *hetero* duplex targets binding of TFO at TFO:duplex ratio 2:1 (upper panel), and 24:1 (lower panel) in the absence of BQQ. For comparison also the *homo* duplex (dupDNA45) target binding of TFO in the presence of BQQ is shown. Error bars show mean + SEM (n = 4 except for 45-D5LD where n = 3).

At all time-points and ratios, the triplex formation in the presence of the triplex-specific intercalating compound BQQ is significantly higher, in accordance with the ability of BQQ to bind and stabilize triplex structures[16]. Interestingly, at the higher ratio there is a trend suggesting that having two or more consecutive LNA nucleotides in the *hetero* duplex reduces the rate of triplex formation, as demonstrated in Fig 6. At ratio 24:1, the 45-1D1L and 45-2D1L *hetero* duplexes more rapidly form triplexes, while for the 45-2L2D and 45-D5LD *hetero* duplexes, the formation rate is somewhat reduced. At the lower ratio, this trend is less pronounced.

We have previously shown that at low ratios and short time points BQQ significantly influences the triplex formation by binding to and stabilizing the triplex once it is formed, rather than affecting the rate of triplex formation [12]. In the present study the same phenomenon is observed, at ratio 24:1 for 0.5 h and 1 h time points, the effect of BQQ in yielding higher triplex formation being significant for all tested sets of duplexes + TFO (S4 Fig).

All together, these experimental findings support the modeling data and highlight the importance of LNA-induced pre-alignment of the duplex conformation to facilitate triplex formation. The results indicate that for TFO binding it is not optimal to have two or more consecutive LNA residues in the *hetero* duplex.

### Triplex conformation and TFO binding

**Fig 7:**
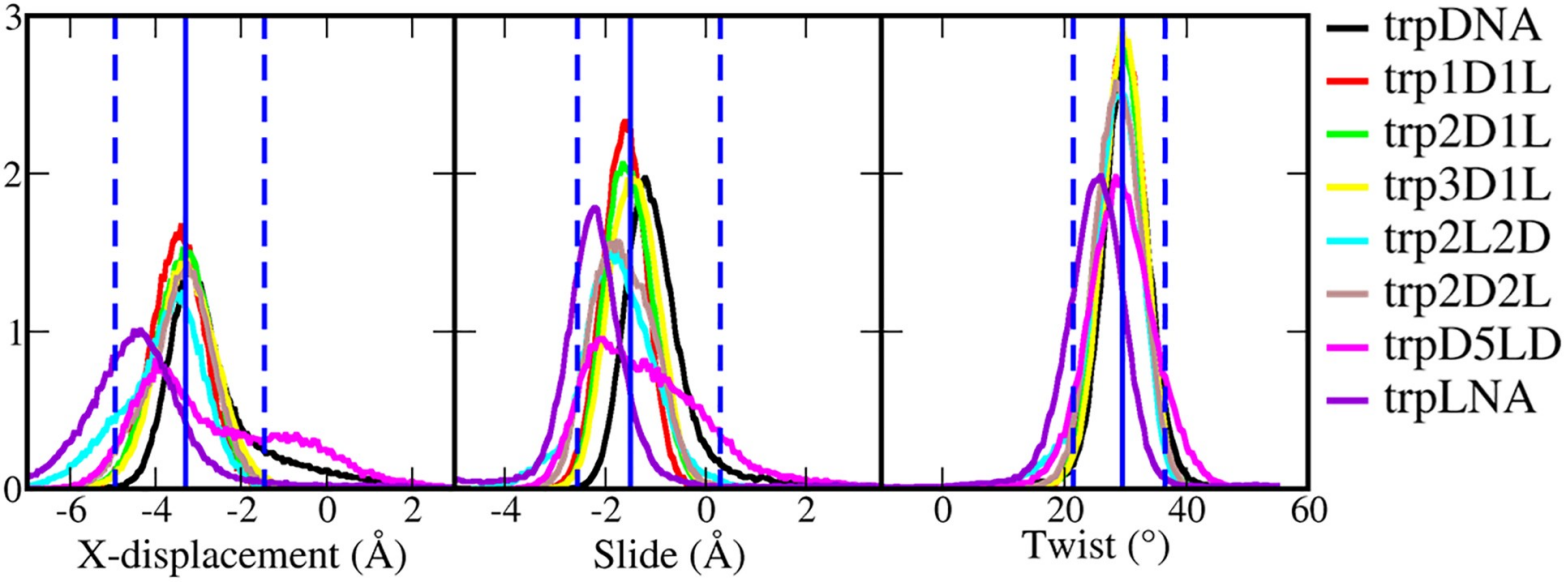
Base pair geometries (x-displacement, slide and twist) for selected triplex structures. The histograms were sampled from 500 ns simulations. The blue vertical lines show the statistical data from trpDNA, trp1D1L, trp2D1L, trp3D1L, trp2D2L and trp2L2D, where the solid line is the average value and dashed lines indicate the range covering the conformation of 95% snapshots.

On average, stable triplex conformations (trpDNA, trp1D1L, trp2D1L, trp3D1L, trp2D2L and trp2L2D) show narrower distribution for helical parameters than the corresponding duplexes with similar helical parameter distribution (Table 3 and Fig 7). This indicates that the conformational requirement to form triplex is more strict than for duplex formation and confirms the choice of the reference values (-3.2 Å for x-displacement, -1.4 Å for slide and 29.5° twist) as an appropriate criterion to define a LirA helix conformation.

**Table 3:** Statistical base pair geometries for the simulated triplexes. Analysis is performed on the following triplexes trpDNA, trp1D1L, trp2D1L, trp3D1L, trp2D2L and trp2L2D. For comparison the parameters of dupDNA and dup1D1L are also reported.

Base pair geometries		Triplex			dupDNA	dup1D1L
		Lower^a^	Average	Upper^a^		
Axis (Å)	X-displacement	-4.95	-3.30	-1.45	-0.77	-4.47
	Y-displacement	-0.95	0.30	1.55	0.14	0.42
Axis (°)	Inclination	-6.0	6.5	18.5	10.2	12.0
	Tip	-6.5	3.0	13.5	2.7	4.4
Intra bp (Å)	Shear	-0.65	0.00	0.60	-0.09	-0.06
	Stretching	-0.25	-0.05	0.25	-0.10	-0.03
	Stagger	-1.25	-0.20	0.80	-0.23	-0.07
Intra bp (°)	Buckle	-24.5	0.5	22.0	4.4	5.1
	Propeller	-23.5	-6.0	13.5	-12.0	-6.9
	Opening	-8.0	2.0	14.0	3.1	2.9
Inter bp (Å)	Shift	-1.45	-0.30	0.75	-0.23	-0.36
	Slide	-2.55	-1.50	-0.30	0.10	-1.77
	Rise	2.65	3.40	4.55	3.41	3.37
Inter bp (°)	Tilt	-13.0	-2.0	12.5	-1.8	-2.3
	Roll	-11.0	3.0	14.0	6.0	6.3
	Twist	21.5	29.5	36.5	33.9	28.6

^a^. The lower and upper bound, within which the 95% area under curve is distributed.

Based on the simulation results, the capability of duplexes to adopt a triplex conformation is: dup1D1L, dup2D1L, dup2D2L/2L2D > dup3D1L > dupDNA. DupD5LD and dupLNA did not maintain stable WC base pairs in the presence of a TFO, and their corresponding triplex conformations clearly diverge from the other triplexes (Fig 7 and S3 Fig). Those two triplexes probably have too high content of LNAs, resulting in an over-unwound helix. The EMSA results show that 45-1D1L and 45-2D1L *hetero* duplexes are equivalent and superior for TFO binding, whereas the *homo* duplex was inferior (Fig 5). 45-2L2D is not as efficient as 45-2D1L but still improves triplex formation compared to 45-D5LD. This indicates that the two consecutive LNA nucleotides may reduce binding affinity, even if they fulfill the conformational criteria. We speculate that consecutive LNAs slow down TFO binding, due to additional conformational adaption upon triplex formation.

In summary, we suggest that a duplex with good capacity to form a triplex needs to have a helical conformation, with x-displacement, slide and twist parameters closer to −3.3 Å, −1.5 Å and 29.5°, respectively, and two consecutive LNA residues or more should be avoided in the polypyrimidine strand.

## Conclusion

We have used molecular dynamics simulation and the electrophoretic mobility shift assay to systematically study the propensity of *hetero* duplexes to form triple-helix structures while having the same sequence but with different LNA/DNA contents in the polypyrimidine strand. All *hetero* duplexes form stable helical structure but with different conformations. The LNA/DNA mixmer oligonucleotides modulate duplex conformation. Duplexes conformationally similar to triple-helix-type conformation show high propensity to bind a TFO. Thus, the combination of simulation and experimental binding assays elucidated that the best strategy to design a duplex with a high triplex propensity is to have a ratio of LNA:DNA of 1:1 or 1:2 and avoiding two or more consecutive LNA nucleotides in the sequence.

The results support a structure-based design strategy for oligonucleotides, where first the mixmers are selected based on their structural features *in silico* and then a selected group is tested *in vitro*. Moreover, the results show that a triplex structure has a more conserved and defined conformation than the corresponding duplex, with narrow distributions of helicoidal parameters and is characterized by a LirA helical conformation.

## Supporting information

S1 Table Average base pair parameters. (PDF)

S1 Fig Resistance of base pairs in duplexes. (PDF)

S2 Fig Resistance of base pairs in triplexes. (PDF)

S3 Fig Selected base pair geometries. (PDF)

S4 Fig The effect of BQQ on triplex formation. (PDF)

S1 File Gels. (PDF)

S2 File Simulation inputs. (TGZ)
