# Supplementary material for: The ability of locked nucleic acid oligonucleotides to pre-structure the double helix: A molecular simulation and binding study
Source: PLoS One. 2019 Feb 12;14(2):e0211651. doi: 10.1371/journal.pone.0211651 (PMC6372149; doi:10.1371/journal.pone.0211651)
Supplement: S1 Table — (PDF) [file pone.0211651.s001.pdf]

Table S1. The average base pair parameters of eight duplexes, counted from 500 ns simulations for each system.

	x-displacement (Å)	Slide (Å)	Twist (°)
dupDNA	-0.77	0.1	33.9
dup1D1L	-4.47	-1.77	28.6
dup2D1L	-2.77	-0.96	31.2
dup3D1L	-2.20	-0.71	32.1
dup2D2L	-3.53	-1.33	29.4
dup2L2D	-3.45	-1.34	29.5
dupD5LD	-2.58	-0.9	30.9
dupLNA	-5.58	-2.18	26
