# Supplementary material for: The ability of locked nucleic acid oligonucleotides to pre-structure the double helix: A molecular simulation and binding study
Source: PLoS One. 2019 Feb 12;14(2):e0211651. doi: 10.1371/journal.pone.0211651 (PMC6372149; doi:10.1371/journal.pone.0211651)
Supplement: S1 Fig — (PDF) [file pone.0211651.s002.pdf]

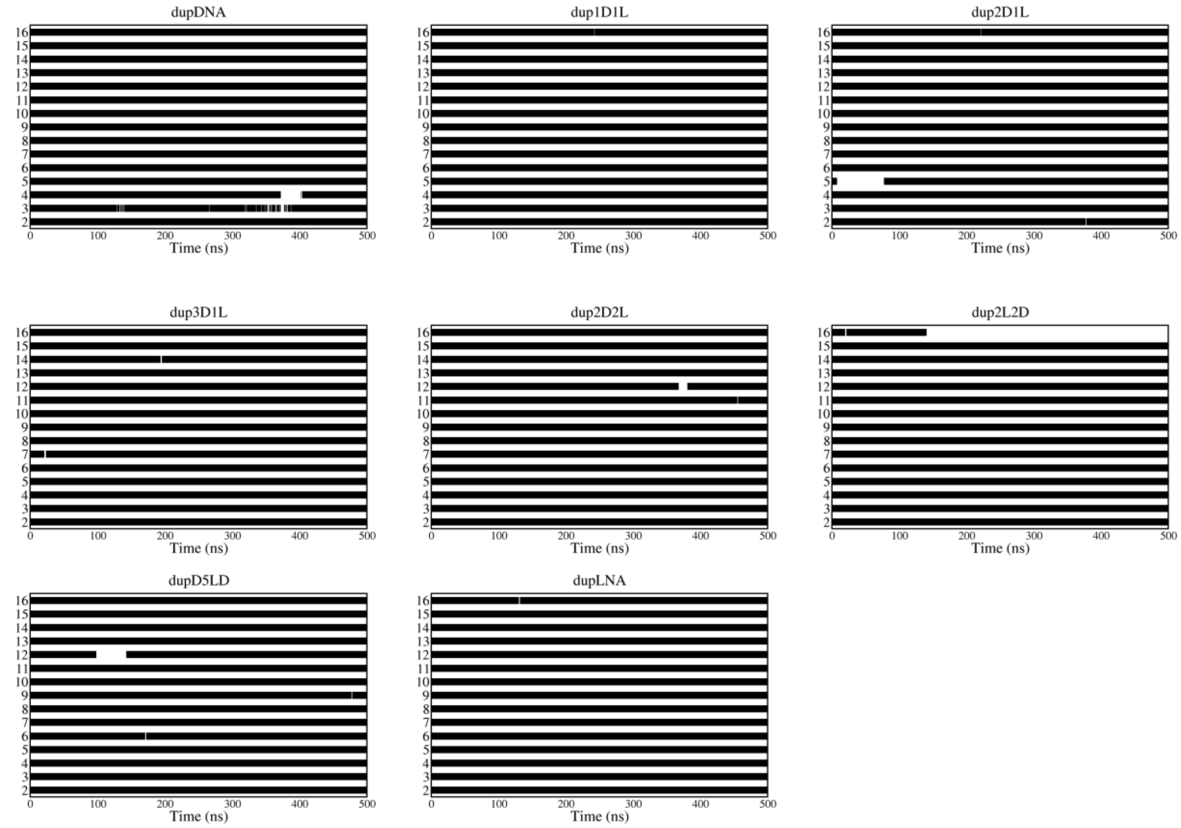

Fig S1. The resistance of base pairs in duplexes as the function of simulation time. The stripes are filled by the snapshots with closed WC base pairs, whereas left blank if the snapshots with opening WC base pairs. The end base pairs (bp.1 & 17) are not shown.
