# Supplementary material for: The ability of locked nucleic acid oligonucleotides to pre-structure the double helix: A molecular simulation and binding study
Source: PLoS One. 2019 Feb 12;14(2):e0211651. doi: 10.1371/journal.pone.0211651 (PMC6372149; doi:10.1371/journal.pone.0211651)
Supplement: S2 Fig — (PDF) [file pone.0211651.s003.pdf]

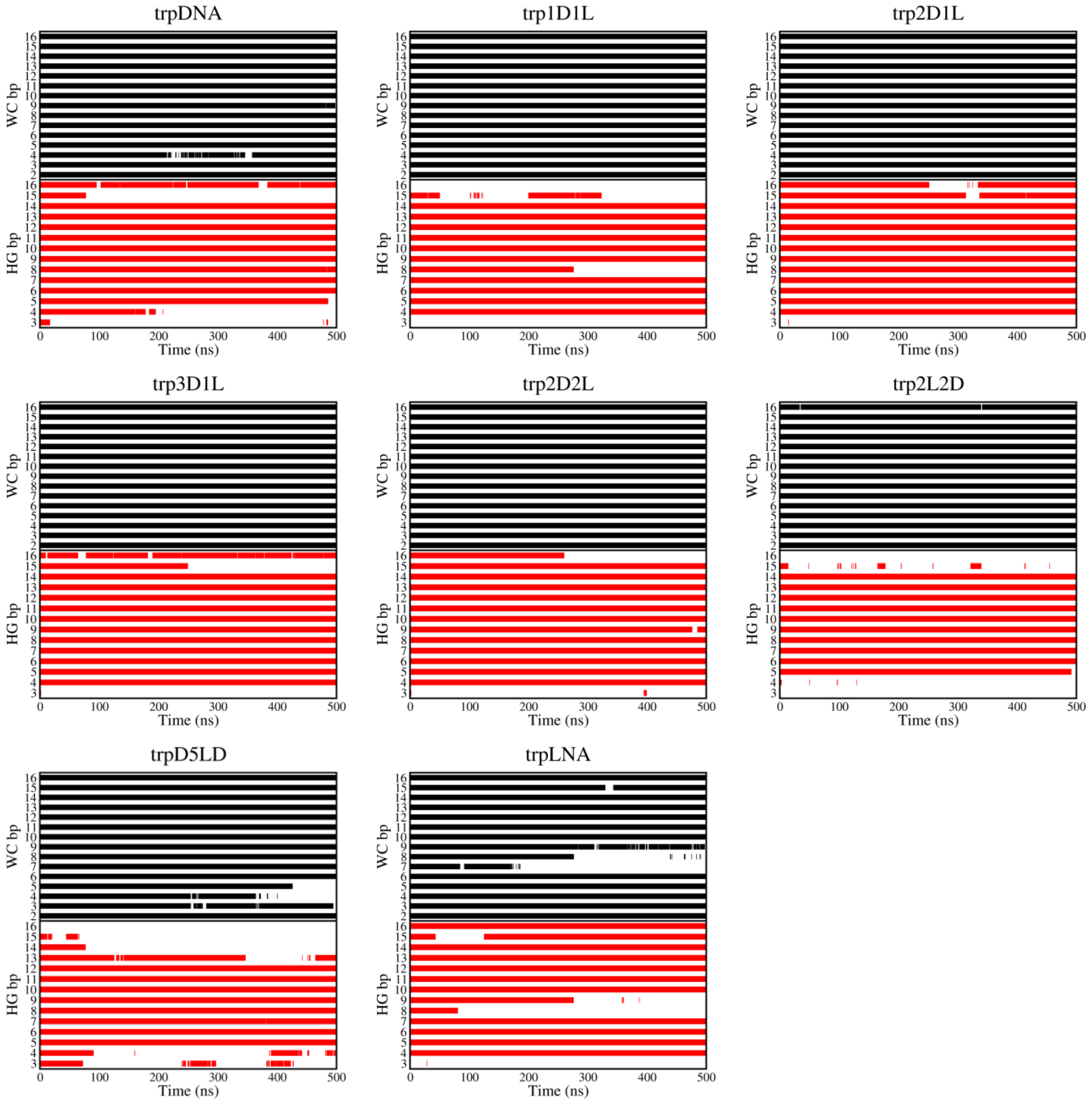

Fig S2. The resistance of base pairs in triplexes as the function of simulation time. The stripes are filled by the snapshots with closed base pairs, whereas left blank if the snapshots with opening base pairs. The end base pairs (bp.1 & 17 of duplex and bp.17 of TFO) are not shown. The Watson-Crick (WC) base pairs are shown in black and the Hoogsteen (HG) base pairs are in red.
