# Supplementary material for: The ability of locked nucleic acid oligonucleotides to pre-structure the double helix: A molecular simulation and binding study
Source: PLoS One. 2019 Feb 12;14(2):e0211651. doi: 10.1371/journal.pone.0211651 (PMC6372149; doi:10.1371/journal.pone.0211651)
Supplement: S3 Fig — (PDF) [file pone.0211651.s004.pdf]

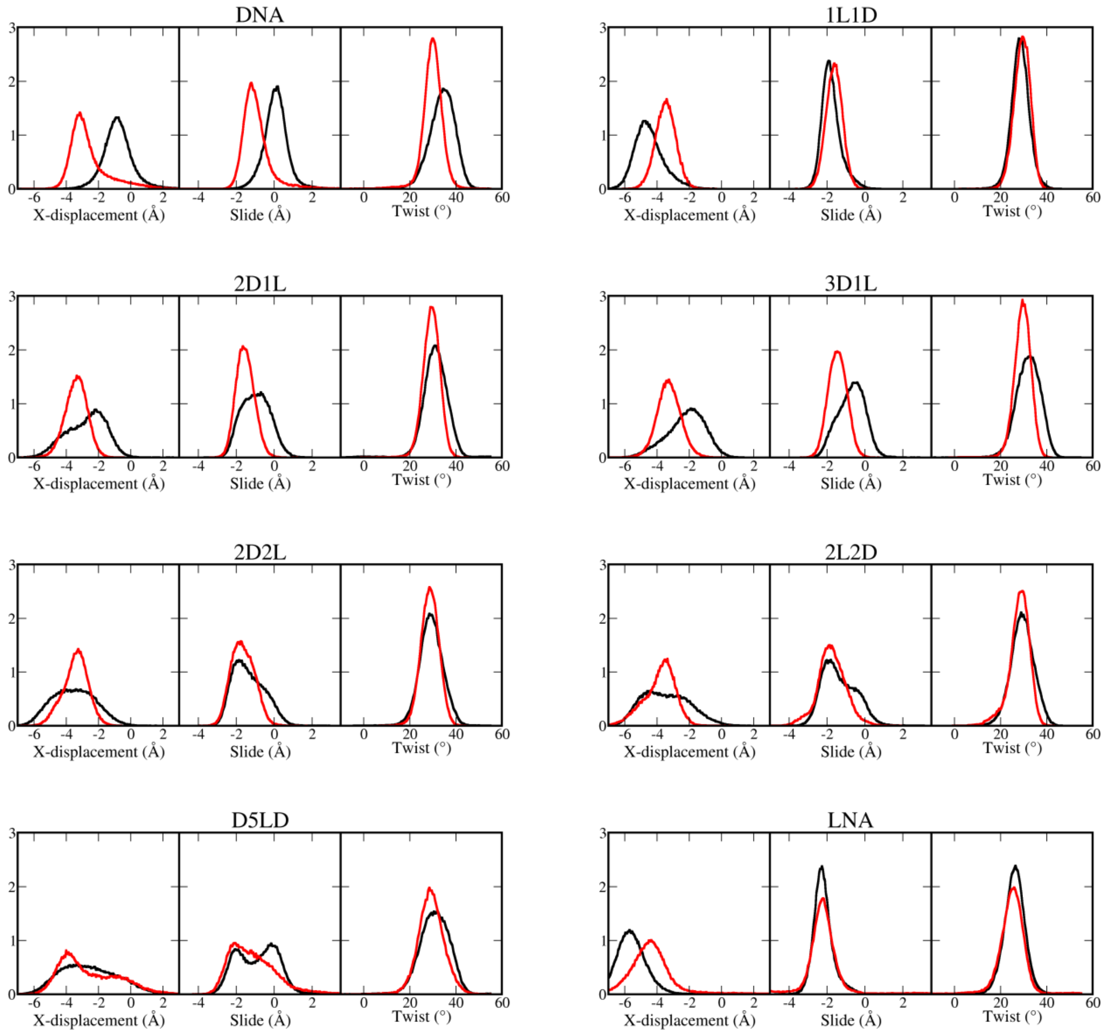


**Fig S3.** The selected base pair geometries (x-displacement, slide and twist) of each duplex and corresponding triplex. The histograms were sampled from 500 ns simulations. The triplex conformations are shown in red and duplexes are in black.
