# Supplementary material for: The ability of locked nucleic acid oligonucleotides to pre-structure the double helix: A molecular simulation and binding study
Source: PLoS One. 2019 Feb 12;14(2):e0211651. doi: 10.1371/journal.pone.0211651 (PMC6372149; doi:10.1371/journal.pone.0211651)
Supplement: S4 Fig — (PDF) [file pone.0211651.s005.pdf]

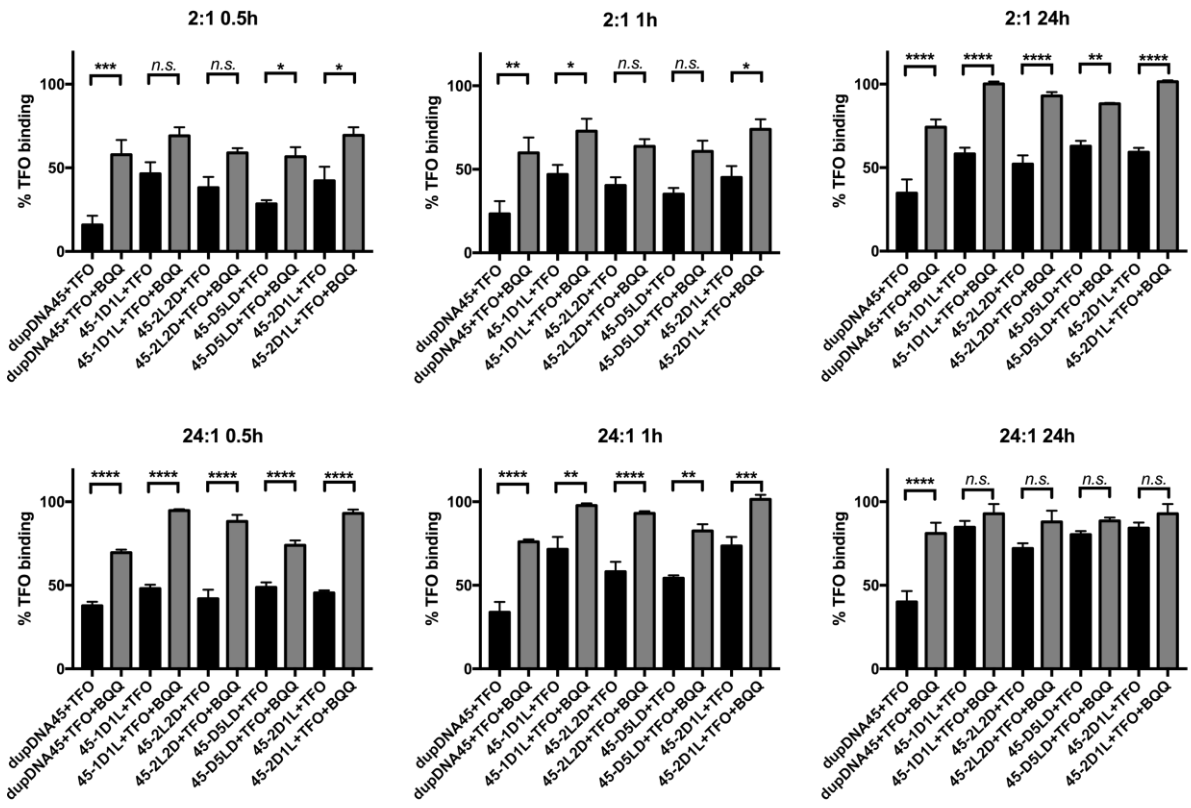

Fig S4. The effect of BQQ on triplex formation. Graphs show the percentage of TFO binding at 0.5, 1 and 24 h respectively at the two ratios studied (2:1 and 24:1 TFO:duplex). For duplex names, see Table 2 and Fig. 4. Significant differences between triplexes formed with or without BQQ are marked with stars (**** $p<0.0001$, *** $p<0.001$, ** $p<0.01$, * $p<0.05$); nonsignificant differences are marked with n.s. Statistical analysis was performed using GraphPad prism (v6) and one-way ANOVA to determine significance. Error bars show the mean+SEM, n=4 for all samples and time points, except for the 45-D5LD samples where n=3.
