# Supplementary material for: The ability of locked nucleic acid oligonucleotides to pre-structure the double helix: A molecular simulation and binding study
Source: PLoS One. 2019 Feb 12;14(2):e0211651. doi: 10.1371/journal.pone.0211651 (PMC6372149; doi:10.1371/journal.pone.0211651)
Supplement: S1 File — (PDF) [file pone.0211651.s006.pdf]

2:1 ratio TFO:heteroduplex
0.5 h

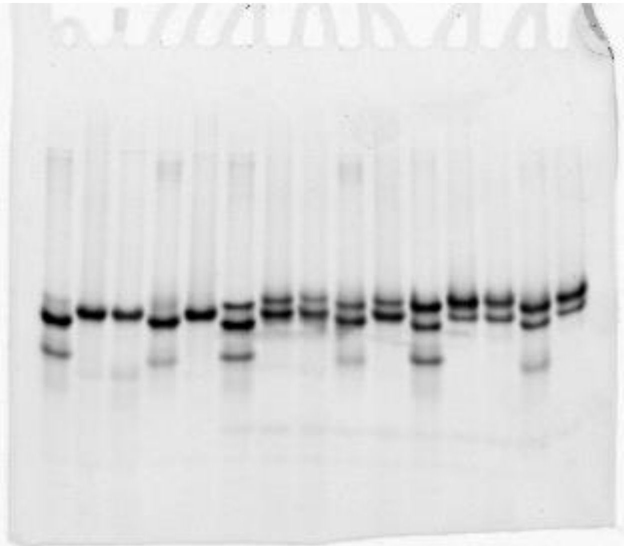

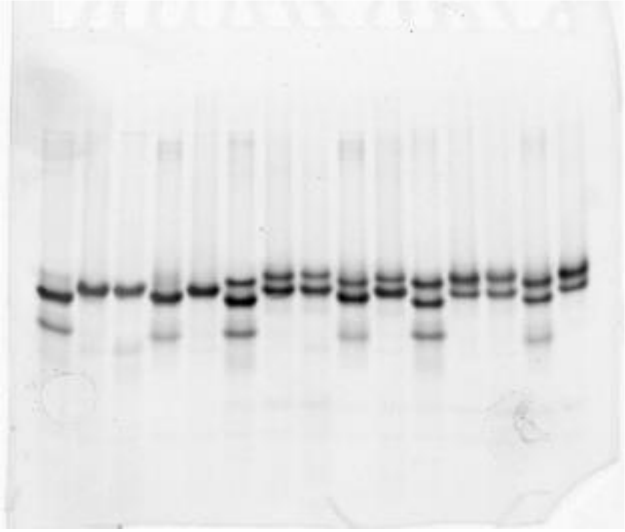

From left to right:
1= dupDNA45
2= 1D1L
3= 2L2D
4= 5midL
5= 2D1L
6= dupDNA45+TFO
7= 1D1L+TFO
8= 2L2D+TFO
9= 5midL+TFO
10= 2D1L+TFO
11= dupDNA45+TFO+BQQ
12= 1D1L+TFO+BQQ
13= 2L2D+TFO+BQQ
14= 5midL+TFO+BQQ
15= 2D1L+TFO+BQQ

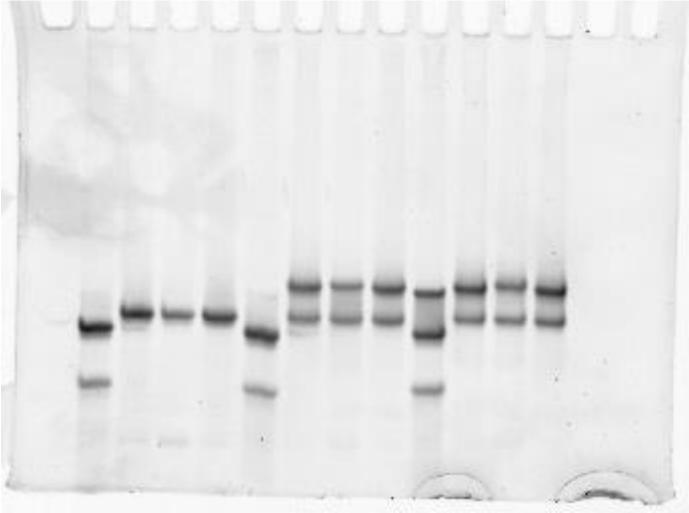

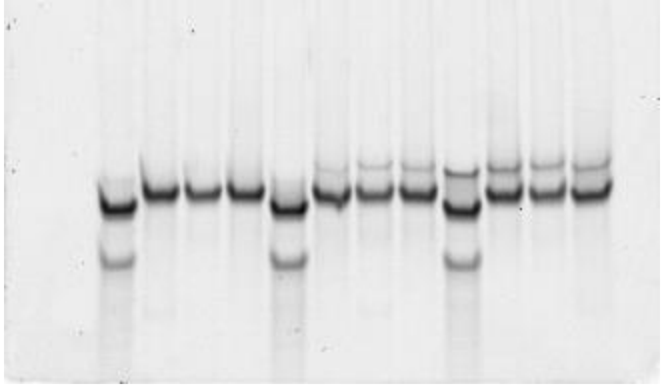

From left to right:
1= dupDNA45
2= 1D1L
3= 2L2D
4= 2D1L
5= dupDNA45+TFO
6= 1D1L+TFO
7= 2L2D+TFO
8= 2D1L+TFO
9= dupDNA45+TFO+BQQ
10= 1D1L+TFO+BQQ
11= 2L2D+TFO+BQQ
12= 2D1L+TFO+BQQ

2:1 ratio TFO:heteroduplex
1 h

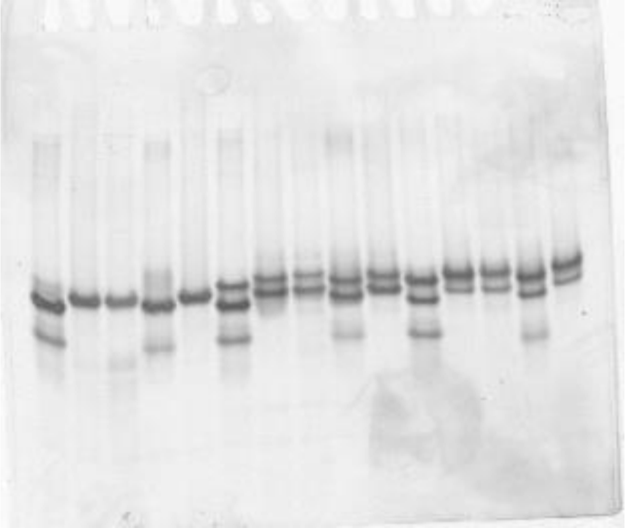

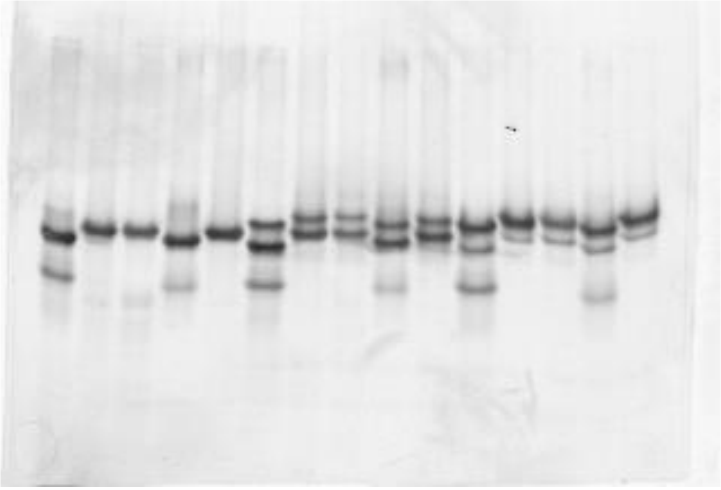

From left to right:
1= dupDNA45
2= 1D1L
3= 2L2D
4= 5midL
5= 2D1L
6= dupDNA45+TFO
7= 1D1L+TFO
8= 2L2D+TFO
9= 5midL+TFO
10= 2D1L+TFO
11= dupDNA45+TFO+BQQ
12= 1D1L+TFO+BQQ
13= 2L2D+TFO+BQQ
14= 5midL+TFO+BQQ
15= 2D1L+TFO+BQQ

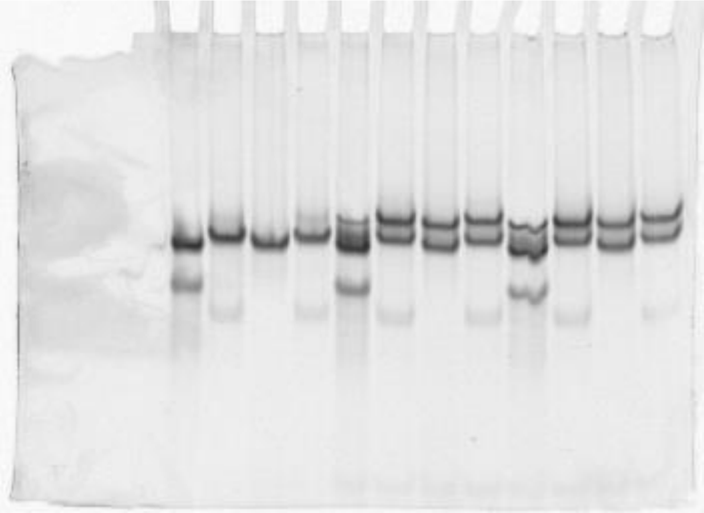

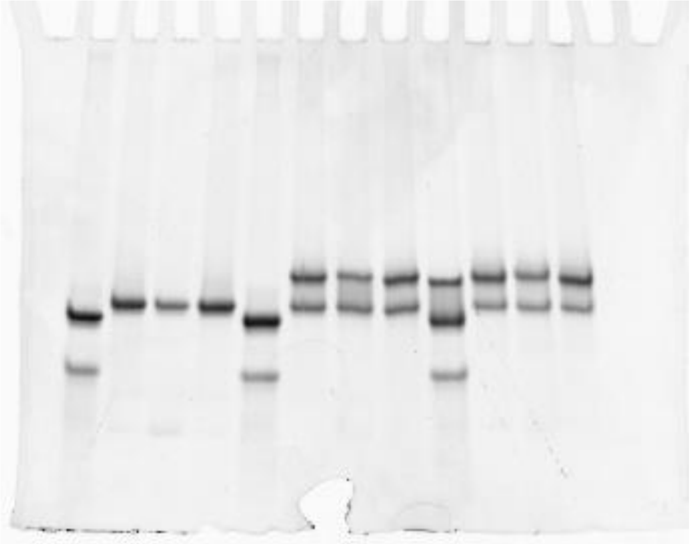

From left to right:
1= dupDNA45
2= 1D1L
3= 2L2D
4= 2D1L
5= dupDNA45+TFO
6= 1D1L+TFO
7= 2L2D+TFO
8= 2D1L+TFO
9= dupDNA45+TFO+BQQ
10= 1D1L+TFO+BQQ
11= 2L2D+TFO+BQQ
12= 2D1L+TFO+BQQ

2:1 ratio TFO:heteroduplex
24 h

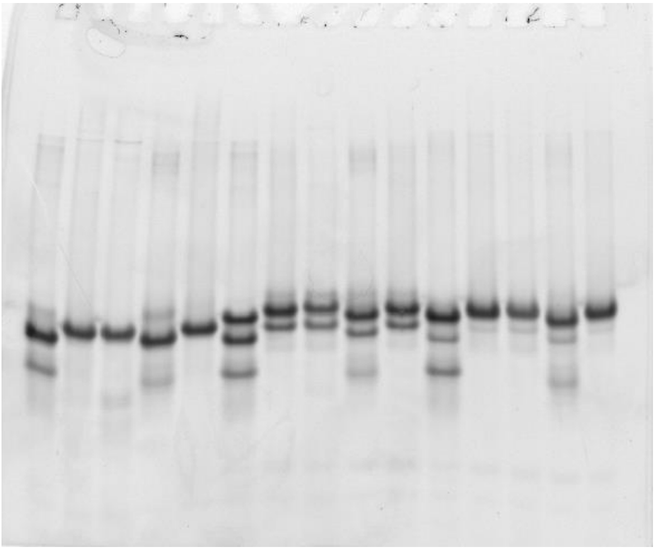

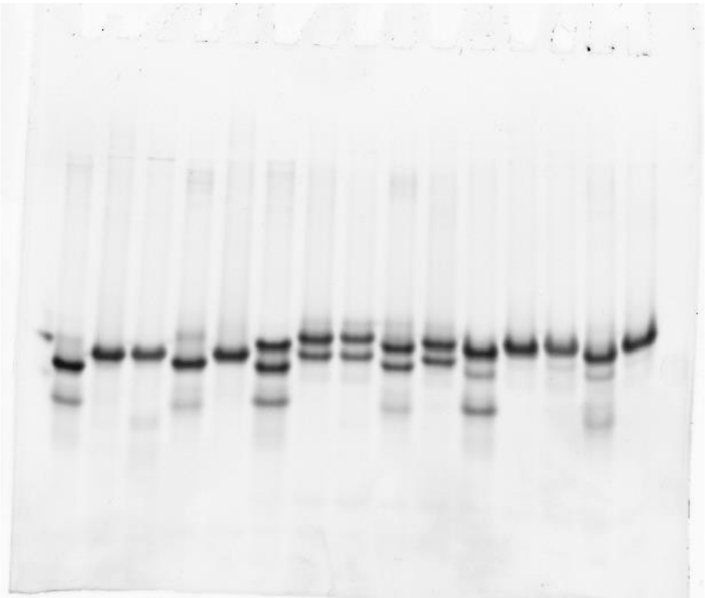

From left to right:
1= dupDNA45
2= 1D1L
3= 2L2D
4= 5midL
5= 2D1L
6= dupDNA45+TFO
7= 1D1L+TFO
8= 2L2D+TFO
9= 5midL+TFO
10= 2D1L+TFO
11= dupDNA45+TFO+BQQ
12= 1D1L+TFO+BQQ
13= 2L2D+TFO+BQQ
14= 5midL+TFO+BQQ
15= 2D1L+TFO+BQQ

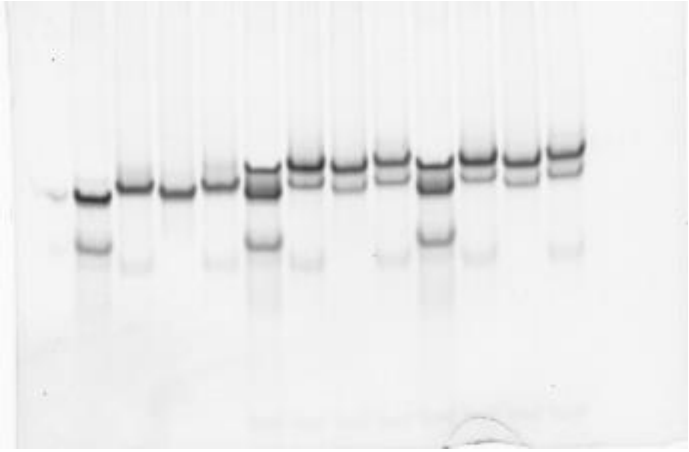

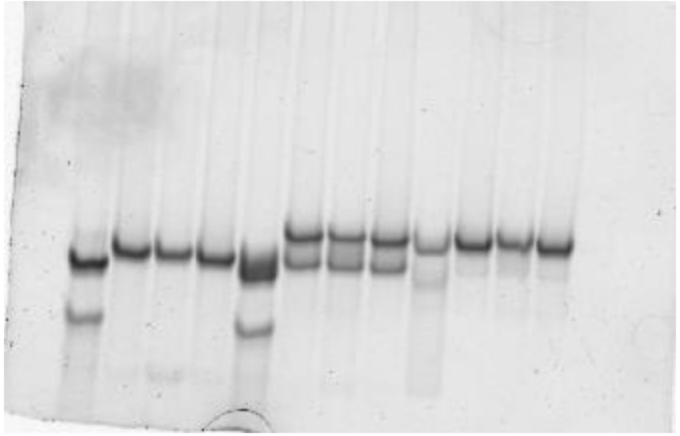

From left to right:
1= dupDNA45
2= 1D1L
3= 2L2D
4= 2D1L
5= dupDNA45+TFO
6= 1D1L+TFO
7= 2L2D+TFO
8= 2D1L+TFO
9= dupDNA45+TFO+BQQ
10= 1D1L+TFO+BQQ
11= 2L2D+TFO+BQQ
12= 2D1L+TFO+BQQ

24:1 ratio TFO:heteroduplex
0.5 h

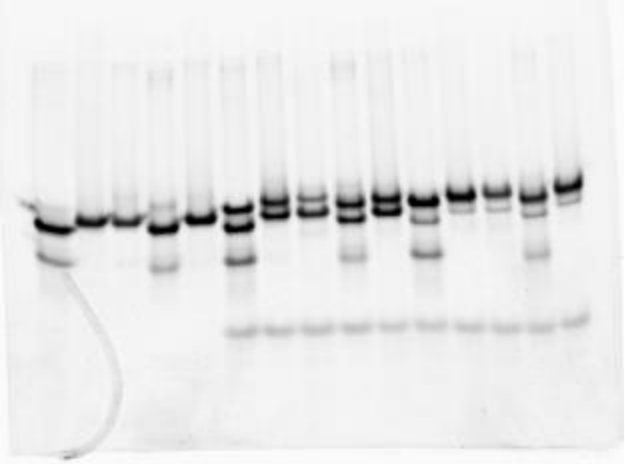

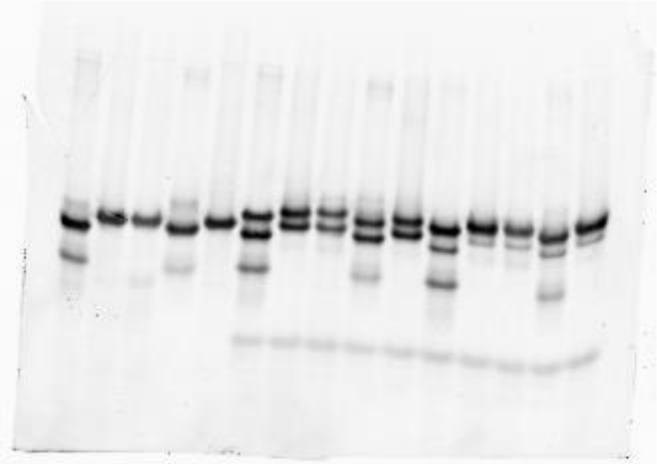

From left to right:
1= dupDNA45
2= 1D1L
3= 2L2D
4= 5midL
5= 2D1L
6= dupDNA45+TFO
7= 1D1L+TFO
8= 2L2D+TFO
9= 5midL+TFO
10= 2D1L+TFO
11= dupDNA45+TFO+BQQ
12= 1D1L+TFO+BQQ
13= 2L2D+TFO+BQQ
14= 5midL+TFO+BQQ
15= 2D1L+TFO+BQQ

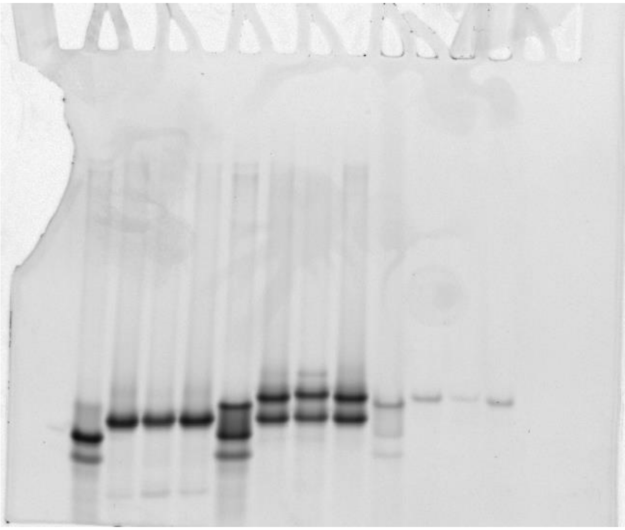

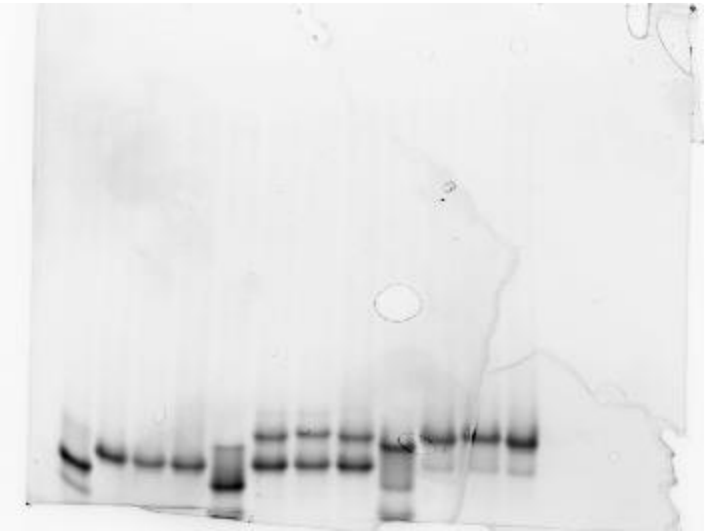

From left to right:
1= dupDNA45
2= 1D1L
3= 2L2D
4= 2D1L
5= dupDNA45+TFO
6= 1D1L+TFO
7= 2L2D+TFO
8= 2D1L+TFO
9= dupDNA45+TFO+BQQ
10= 1D1L+TFO+BQQ
11= 2L2D+TFO+BQQ
12= 2D1L+TFO+BQQ

24:1 ratio TFO:heteroduplex
1 h

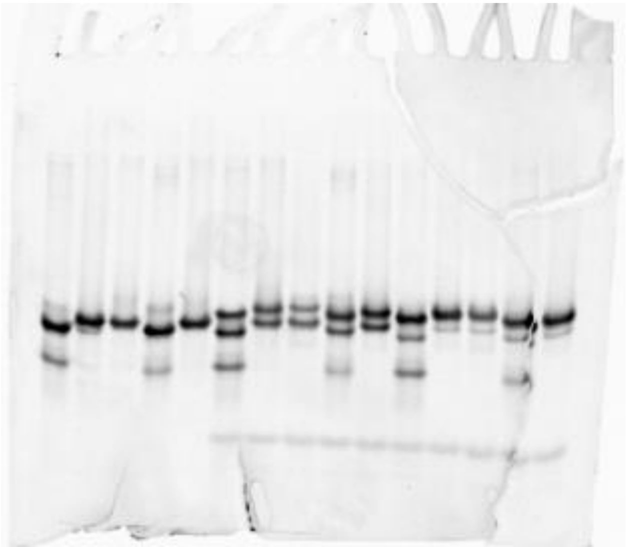

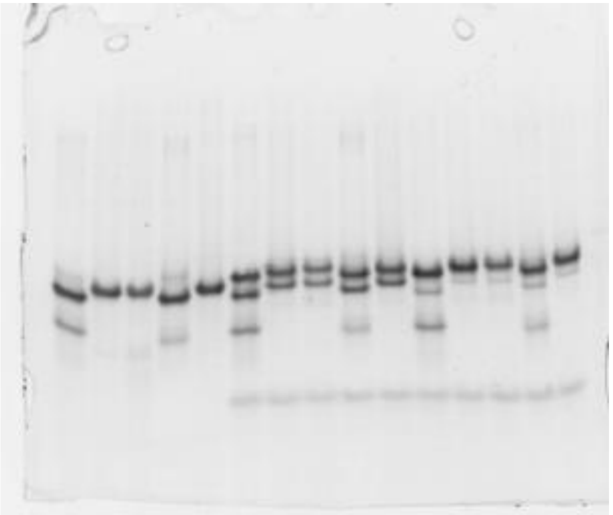

From left to right:
1= dupDNA45
2= 1D1L
3= 2L2D
4= 5midL
5= 2D1L
6= dupDNA45+TFO
7= 1D1L+TFO
8= 2L2D+TFO
9= 5midL+TFO
10= 2D1L+TFO
11= dupDNA45+TFO+BQQ
12= 1D1L+TFO+BQQ
13= 2L2D+TFO+BQQ
14= 5midL+TFO+BQQ
15= 2D1L+TFO+BQQ

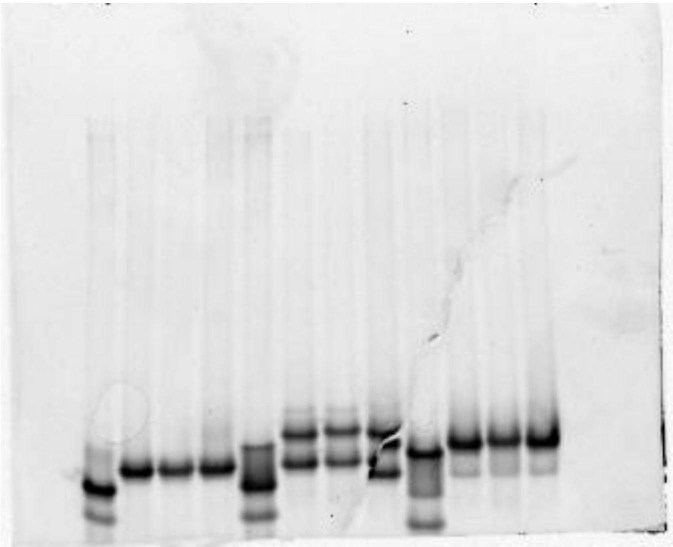

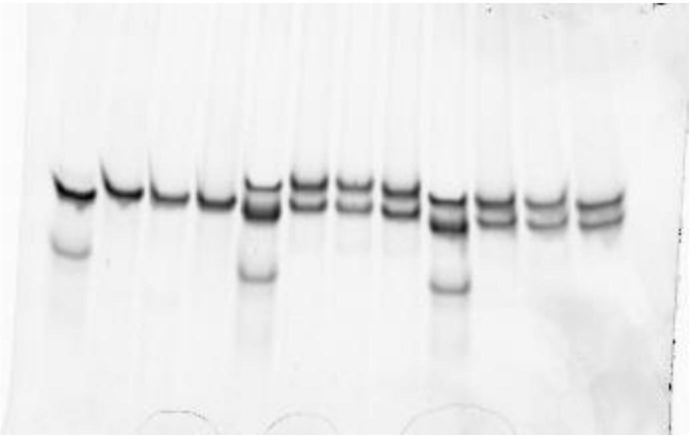

From left to right:
1= dupDNA45
2= 1D1L
3= 2L2D
4= 2D1L
5= dupDNA45+TFO
6= 1D1L+TFO
7= 2L2D+TFO
8= 2D1L+TFO
9= dupDNA45+TFO+BQQ
10= 1D1L+TFO+BQQ
11= 2L2D+TFO+BQQ
12= 2D1L+TFO+BQQ

24:1 ratio TFO:heteroduplex
24 h

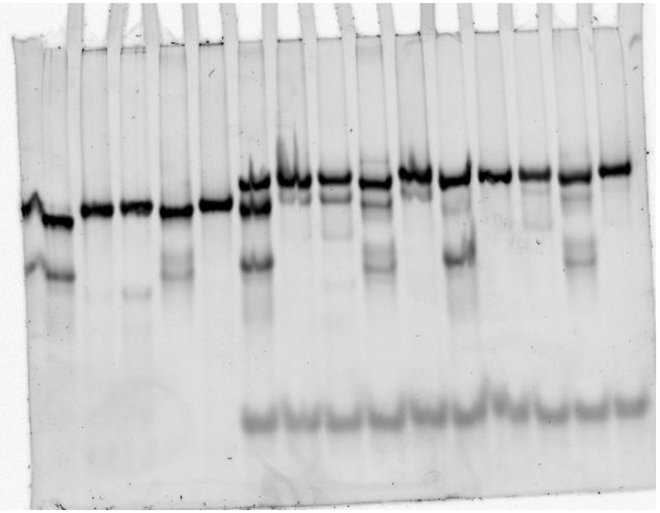

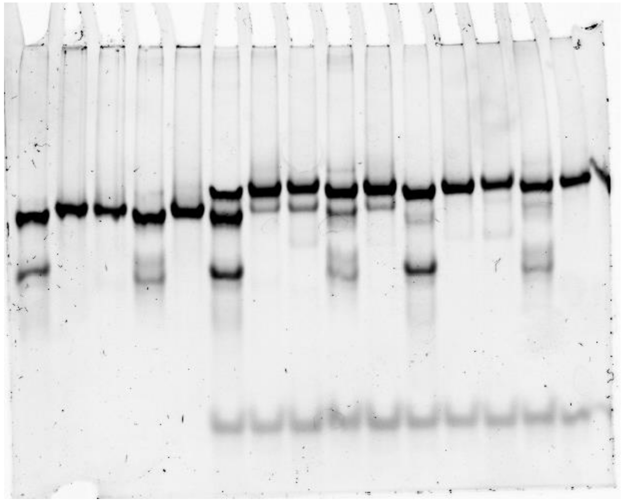

From left to right:
1= dupDNA45
2= 1D1L
3= 2L2D
4= 5midL
5= 2D1L
6= dupDNA45+TFO
7= 1D1L+TFO
8= 2L2D+TFO
9= 5midL+TFO
10= 2D1L+TFO
11= dupDNA45+TFO+BQQ
12= 1D1L+TFO+BQQ
13= 2L2D+TFO+BQQ
14= 5midL+TFO+BQQ
15= 2D1L+TFO+BQQ

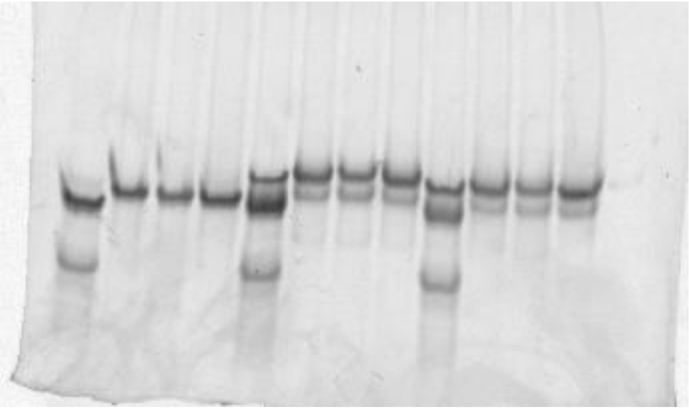

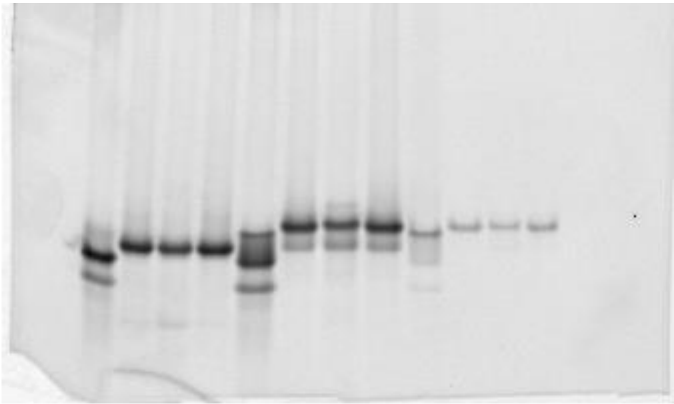

From left to right:
1= dupDNA45
2= 1D1L
3= 2L2D
4= 2D1L
5= dupDNA45+TFO
6= 1D1L+TFO
7= 2L2D+TFO
8= 2D1L+TFO
9= dupDNA45+TFO+BQQ
10= 1D1L+TFO+BQQ
11= 2L2D+TFO+BQQ
12= 2D1L+TFO+BQQ

5midL extra time points

2:1 ratio TFO:heteroduplex

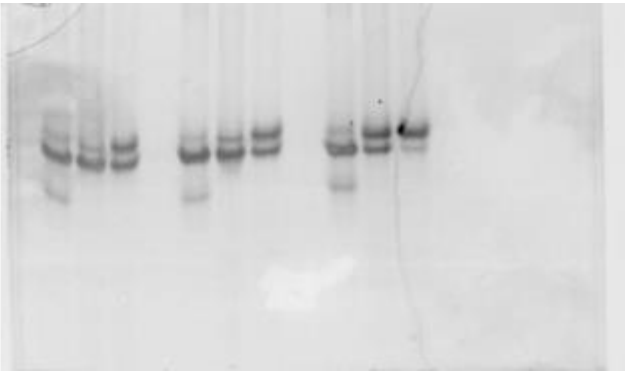

From left to right:
1= 5midL 0.5h
2= 5midL+TFO 0.5h
3= 5midL+TFO +BQQ 0.5h

4= 5midL 1h
5= 5midL+TFO 1h
6= 5midL+TFO+BQQ 1h

7= 5midL 24h
8= 5midL+TFO 24h
9= 5midL+TFO+BQQ 24h

24:1 ratio TFO:heteroduplex

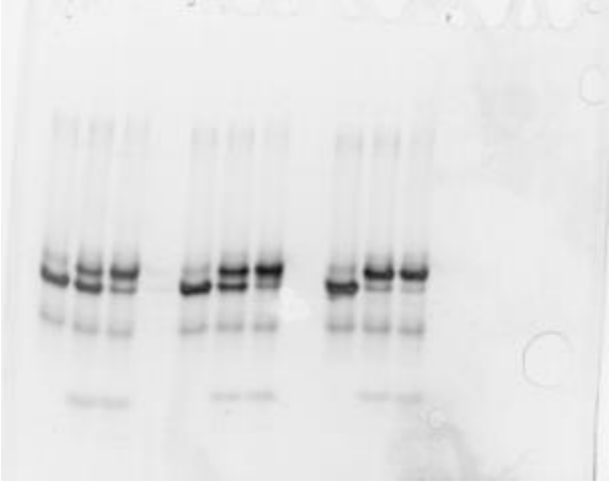

From left to right:
1= 5midL 0.5h
2= 5midL+TFO 0.5h
3= 5midL+TFO +BQQ 0.5h

4= 5midL 1h
5= 5midL+TFO 1h
6= 5midL+TFO+BQQ 1h

7= 5midL 24h
8= 5midL+TFO 24h
9= 5midL+TFO+BQQ 24h
